# A study on the effects of college students’ knowledge-sharing behavior on group performance and individual social status

**DOI:** 10.3389/fpsyg.2023.1172554

**Published:** 2023-06-16

**Authors:** Li Jia, Zhikun Liang, Chuanping Lei, Li Huang

**Affiliations:** ^1^College of Humanities and Management, Guangdong Medical University, Dongguan, China; ^2^School of Education, South China Normal University, Guangzhou, China; ^3^School of Marxism, Sun Yat-sen University, Guangzhou, China

**Keywords:** university students, knowledge sharing, group performance, individual status, appreciation for the sharer

## Abstract

**Introduction:**

Universities, as typical knowledge-based organizations, engage in various knowledge management activities, including knowledge acquisition, storage, application, and innovation. This research focuses on applying organizational knowledge management principles to college student groups in universities, aiming to explore the current state of knowledge-sharing behaviors within these groups and investigate the relationship between group performance, individual social status, and knowledge-sharing behaviors.

**Methods:**

A sample of 497 college students from six universities in China was randomly selected, and an econometric analysis using structural equation modeling was conducted with SPSS21.0 and AMOS21.0 to examine their knowledge-sharing behaviors, individual social status, and group performance.

**Results:**

The findings reveal that individual knowledge-sharing behavior significantly influences the knowledge sharing behavior of others and the recognition received by the sharer. Moreover, the knowledge-sharing behavior of others positively contributes to group performance, while recognition from others enhances the social status of the sharer. Furthermore, the knowledge-sharing behaviors of others mediate the relationship between individual knowledge-sharing behaviors and group performance, while others’ recognition of the sharer mediates the relationship between individual knowledge-sharing behaviors and the sharer’s social status. This study provides valuable theoretical guidance for organizational knowledge management and the development of college students’ learning abilities, establishing a crucial foundation for comprehensive, scientific, and standardized student management.

**Conclusion:**

Overall, this research contributes to understanding the dynamics of knowledge sharing among college students and highlights the importance of incorporating knowledge management principles in educational settings. The findings underscore the positive impact of knowledge sharing on group performance and individual social status, emphasizing the need for effective knowledge sharing practices to enhance student management in higher education institutions.

## 1. Introduction

In an organization, the quantity and quality of knowledge owned by different knowledge subjects vary due to their talents, qualities, efforts, opportunities, and other factors. Knowledge sharing refers to the process by which knowledge owners share knowledge with knowledge demanders through various communication methods to realize knowledge transfer and thus achieve knowledge growth (Lee, 2001; Wang, 2020). In the era of the knowledge economy, knowledge-sharing mutual learning among college students has become a more important topic, which can help college students meet the challenges of increasingly integrated knowledge (Xu et al., 2021). Knowledge-sharing mutual learning of college students can, firstly, enable college students to obtain more knowledge from others’ knowledge, thus broadening their vision, increasing students’ knowledge stock, and effectively improving their learning efficiency. Secondly, it can also transform personal knowledge into collective knowledge, integrate practical resources, improve the knowledge system, and maximize the value of knowledge (Cao, 2017). In addition, knowledge-sharing mutual learning also helps cultivate college students’ independent thinking ability and enhance their team cooperation ability. College students can better learn how to collaborate through mutual communication and sharing to better exert their team cooperation ability (Cheruvelil et al., 2014). Therefore, it is essential to study the knowledge-sharing behavior of college students to improve students’ learning efficiency, enhance learning quality, cultivate students’ innovative thinking, stimulate learning vitality, promote the sharing of learning resources, improve students’ comprehensive ability, enhance students’ social responsibility, and cultivate excellent talents for society.

Knowledge-sharing behavior is an activity that requires the participation of multiple parties. As a sharing subject, the relationship between people can significantly impact knowledge sharing (Dirks and Skarlicki, 2004). However, knowledge sharing does not happen naturally. What factors influence college students’ shared enjoyment of knowledge? It has been found that interpersonal trust is one of the critical factors that determine individuals’ initiative to share knowledge. It refers to an emotional expression of holding positive expectations of others’ behavior in interactions with others. The higher the interpersonal trust between individuals, the more favorable it is to stimulate knowledge-sharing behavior. In addition, the higher the level of interpersonal trust among subjects, the easier it is to establish friendly communication and share a relationship with timely feedback. This friendly sharing relationship can reduce the sense of crisis of “not being able to cover the expenses” of knowledge, enhance the willingness to share knowledge, and thus improve the level of sharing behavior (Gan et al., 2012). In addition to personal factors such as interpersonal trust, the level of individual knowledge sharing is also influenced by the environment. The class climate is a common psychological trait of members in the classroom environment. It is a concentrated expression of the sense of belonging (Wang et al., 2013). The class is the primary environment in which students engage in knowledge learning, and the better the class climate, the higher the level of knowledge sharing (Goh, 2002). The social cognitive theory defines human behavior as the result of the interaction of individual and environmental factors, and environmental factors usually show a role between individual factors and personal behavior (Zhang and Wu, 2016). Although people are increasingly interested in the relationship between knowledge-sharing and group performance, few studies have tried to reveal the impact of the knowledge-sharing mechanism on group performance. Therefore, the impact of individual knowledge-sharing behaviors on group performance and individual social status is still unclear. In particular, in the case of multiple participants in the knowledge-sharing process, the mechanisms of others’ knowledge-sharing behaviors on individual knowledge-sharing and group performance, as well as others’ appreciation and others’ perception of individual knowledge-sharing and individual status are yet to be clarified due to the differences in individual behaviors and individual factors. Based on this, this study intends to address the critical questions as follows.

(1) Whether individual knowledge sharing among college students affects group performance and individual status of sharers.
(2) The role of others’ knowledge sharing in the relationship between individual knowledge sharing and group performance of college students.
(3) The role of others’ appreciation of the sharer in the relationship between individual knowledge sharing and the individual status of the sharer.

This study constructs a mediation model based on structural equations for the relationships of the variables involved. It explores the mediating role played by others’ appreciation and others’ knowledge sharing in the influence of individual knowledge sharing on group performance. Knowledge sharing is an essential driver of performance and status. They affect college student group performance and individual status in different ways. They may have significant implications for college student education in domestic Chinese universities. Answering these questions has essential theoretical guidance value for organizational knowledge management and college students’ learning capacity development, as it can help us better understand the mechanisms of group learning and thus better improve group performance. In addition, the study can provide college students with effective learning strategies to improve their learning efficiency and performance.

## 2. Theories and hypotheses

In recent years, the impact of knowledge-sharing behavior on group performance and individual social status has received increasing attention. In this paper, we review the research in this area to gain insight into the effects of knowledge-sharing behavior on group performance and individual social status.

### 2.1. Effects of knowledge-sharing behavior on group performance

Generally, individual team members need to gain all the knowledge needed for a project, so additional information must be obtained before productive work can be completed (Lin, 2007). Studies have provided insight into knowledge-sharing behavior, analyzing individual knowledge-sharing behavior and its impact on group performance and individual social status from different perspectives. On the one hand, Liao (2008) pointed out that the theme of knowledge sharing is the minimalist formalism of knowledge exchange, supporting the behavioral architecture of knowledge exchange and demonstrating the benefits of using knowledge to increase interaction predictability through empirical evaluations. Stupnisky et al. (2018) analyzed individual knowledge-sharing behavior from the perspective of self-determination theory (SDT) and explored its relationship with individual motivation and autonomy (Wu et al., 2021). Hung et al.’s (2011) study investigated the effects of intrinsic motivation (altruism) and extrinsic motivation (financial rewards, reputational feedback, and reciprocity) on knowledge sharing in group meetings, including the number of ideas generated, idea usefulness, idea creativity, and meeting satisfaction.

On the other hand, Calvó-Armengol and Jackson (2009) modeled the organization as a game in which all intelligence share a typical decision problem, and a certain degree of coordination is required between individual behaviors. Razak et al. (2016) revealed the factors that influence individual knowledge-sharing behavior by analyzing the nature of knowledge-sharing and the theory behind knowledge-sharing behavior (Razmerita et al., 2016). Nguyen (2020) applied two psychological models, the Theory of Rational Behavior (TRA) and the Theory of Planned Behavior (TPB), to predict the development of knowledge-sharing attitudes, intentions, and behaviors. In addition, Chen and Kuo’s (2017) and Pereira and Mohiya’s (2021) study explored the effects of positive and negative employee personal intentions and positive and negative organizational support on knowledge-sharing behavior in the context of knowledge-sharing and hiding. Thus, by observing and analyzing the existing studies, we can see that knowledge-sharing behavior has a vital role in facilitating knowledge flow, collaboration, and innovation in teams and organizations. Individual knowledge-sharing behaviors can stimulate positive responses from others and encourage them to share their knowledge and experiences actively, thus creating a virtuous knowledge-sharing cycle. Such positive interactions help strengthen teamwork and creativity, ultimately enhancing group performance and the social status of individuals. We propose the following hypothesis:

*H1*: Individual knowledge-sharing behavior has a significant positive impact on the knowledge-sharing behavior of others.

A knowledge-sharing perspective provides new perspectives for organizations to promote knowledge-sharing among employees and stimulate their desire to work (Wu et al., 2012; Li et al., 2022). The personal factors that make employees hide their knowledge are lack of knowledge sharing, internal competition, and psychological entitlement rewards (Leijen et al., 2014). Chen et al. (2021) explored the mediating role of knowledge sharing (knowledge collection and knowledge donation) in the relationship between psychological capital and innovative work behavior and found that knowledge collection and knowledge donation play a mediating chain role between psychological capital and innovative work behavior. In addition, knowledge collection has an independent mediating role. Zhou (2022) empirically investigated 310 music-learning students from various universities using sampling techniques and data collected through a structured questionnaire method. Guo (2022) tested a model of factors influencing the knowledge-sharing behavior of online community members using PLS-SEM. Chen et al. (2018) aimed to explore the relationship between organizational innovation climate (OIC) and innovative work behavior (IWB), using psychological safety (P.S.) and knowledge sharing (K.S.) as mediating variables, and found that others’ knowledge-sharing behavior can increase team cohesion and innovation to a certain extent. The following hypotheses were therefore made.

*H2*: Knowledge sharing behavior of others has a mediating role in the relationship between individual knowledge-sharing behavior on group performance. Personal knowledge-sharing behavior will promote the knowledge-sharing behavior of others within the group, which in turn leads to positive group performance.

### 2.2. Impact of knowledge sharing behavior on individual social status

**Figure 1:**
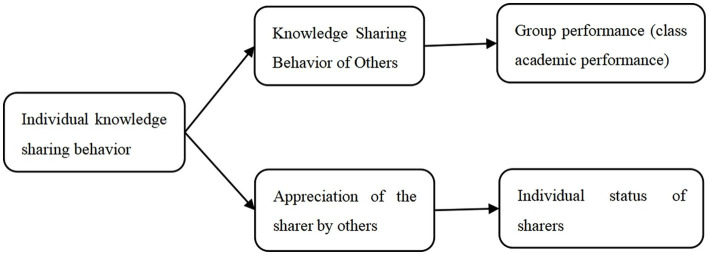
Relationship diagram of study variables.

The Theory of Planned Behavior states that individual behavioral factors that lead to the role of others’ intentions can indirectly influence others’ individual behavior. Some researchers have found that users can generate sociological theoretical exchange for knowledge-sharing behavior in the virtual community environment to achieve a two-way pleasure phenomenon. Based on the political performance appraisal perspective, managers’ appreciation and affirmation of employees’ political behaviors in the performance appraisal process can effectively enhance employees’ motivation (Zhao, 2012). Concerning the social identity theory, it is proposed that individuals increase their self-esteem by achieving or maintaining a positive social identity and that positive self-esteem comes from favorable comparisons between the in-group and the relevant out-group (Meng, 2017). Zhang and Jun (2022) experimentally found that high emotional evaluators are effective in teamwork to enhance the social status of essential team members and form teamwork. Therefore the following hypothesis was made (Figure 1).

*H3*: Individual knowledge-sharing behavior significantly positively affects others' appreciation of the sharer.

*H4*: Appreciation of the sharer by others has a mediating role in the relationship between individual knowledge-sharing behavior on the individual status of the sharer. Individual knowledge-sharing behavior drives others' appreciation of the sharer, which in turn positively influences the sharer's individual status.

## 3. Research subjects and methods

### 3.1. Research subjects

This paper takes Chinese college students as the research sample; using random sampling, 552 questionnaires were distributed to 6 colleges and universities, 552 questionnaires were collected, 497 valid questionnaires were returned, and the effective rate was 90%. Among them, 217 were males, accounting for 43.6% of the total. There were 222 females, accounting for 56.4% of the total number. The education level is generally a bachelor’s degree or above, and the monthly family income is between 3,000–4,000 RMB.

### 3.2. Research methodology

#### 3.2.1. Questionnaire

The questionnaires were analyzed and processed by data analysis with the help of analysis software spss version 21.0 to calculate the reliability and validity test values of the survey scale, as shown in Table 1, the results of the test analysis can be concluded that the cumulative variance interpretation rate of each quantity is greater than 60%. The reliability coefficient is higher than 0.90, which can be obtained from the table with relatively good reliability.

**Table 1:** Values of reliability and validity tests of the scale.

	Individual knowledge-sharing behavior	Knowledge-sharing behavior of others	Appreciation of the sharer by others	Group performance	The individual status of the sharer
Cumulative variance explained	70.526%	76.478%	80.249%	67.842%	66.254%
Overall confidence coefficient	0.877^**^	0.929^**^	0.920^**^	0.881^**^	0.906^**^

** Indicates *p* < 0.01, * indicates *p* < 0.05.

#### 3.2.2. Mathematical statistics

The questionnaire results were statistically and mathematically analyzed in this study, and the data were post-processed using SPSS 21.0 software. Among them, correlation analysis, such as multiple regression analysis, was adopted to analyze the relationship between individual knowledge-sharing behavior, group performance, and individual status and to test the mediating role of others’ knowledge sharer behavior and appreciation of sharers. AMOS 21.0 software was used to validate the structural validity of the conceptual framing model. The contemporary Bootstrap method is the more agreed method to test the mediating effect. The root of the Bootstrap method is the analysis of the correlation of a*b. On the one hand, the Sobel test is taken. In the Sobel test, the data requirements are high, requiring a large sample size and a normal distribution, so the efficiency of this testing method is low. On the other hand, it is the sampling test method for the original sample. The Bootstrap sampling method is a more mainstream test because of its efficiency, and there is no restriction on the distribution pattern of the mediating effect sampling method. The bootstrap sampling method is based on the repeated sampling of the original sample, and the significance of the coefficient of the mediating effect is tested by the 95% confidence interval (CI).

## 4. Results

### 4.1. Discriminant validity test

The results of the mean, standard deviation, and correlation coefficient analysis of each variable are shown in Table 2. from the above table. It is clear that individual knowledge-sharing behavior has a significant positive relationship with others’ knowledge-sharing behavior (*r* = 747, *p* < 0.01), others’ appreciation of sharers (*r* = 758, *p* < 0.01), group performance (*r* = 697, *p* < 0.01), and individual status of sharers (*r* = 738, *p* < 0.01); individual knowledge sharing behavior has a significant positive correlation was found between others’ knowledge sharing behavior and others’ appreciation of the sharer (*r* = 811, *p* < 0.01), group performance (*r* = 738, *p* < 0.01), and the sharer’s individual status (*r* = 770, *p* < 0.01); a significant positive correlation was found between others’ appreciation of the sharer and group performance (*r* = 735, *p* < 0.01), and the sharer’s individual status (*r* = 782, *p* < 0.01). Individual status (*r* = 782, *p* < 0.01); group performance was significantly and positively correlated with the sharer’s status (*r* = 840, *p* < 0.01). These results laid the foundation for subsequent structural equation modeling and hypothesis testing.

**Table 2:** Discriminant validity tests among the variables.

Variables	Average value	Standard deviation	1	2	3	4	5	6	7	8	9
Gender	1.563	0.496	1								
Age	3.115	0.493	−0.034	1							
Literacy	3.771	0.435	0.058	−225**	1						
Monthly family income	4.392	1.433	−0.073	0.153**	−0.176**	1					
Individual knowledge-sharing behavior	4.068	0.492	−0.004	0.244**	−216**	0.258**	1				
Knowledge sharing behavior of others	3.939	0.641	−0.048	0.258**	−302**	0.254**	0.747**	1			
Appreciation of the sharer by others	3.897	0.642	−0.036	0.255**	−0.341**	0.308**	0.758**	0.811**	1		
Group performance	3.971	0.654	−0.012	0.210**	−0.237**	0.243**	0.697**	0.738**	0.735**	1	
Individual status of sharers	3.965	0.640	−0.04	0.282**	−0.252**	0.256**	0.738**	0.770**	0.782**	0.840**	1

*N* = 497, * denotes *p* < 0.05, ** denotes *p* < 0.01.

### 4.2. Reliability and convergence tests

The analysis results in Table 3 show that the standard factor loadings of all variables are more significant than 0.7, indicating that the measurement question items can represent the latent variables well. The combined reliability C.R.S were all greater than the criterion of 0.7; the average method extract AVEs were all greater than the criterion of 0.5, indicating that the scale has good convergent validity.

**Table 3:** Reliability and convergent validity tests for sample data.

Dimensionality	Title	Standardized factor loadings	Clonpa Ha factor	Component reliability	Convergent validity AVE
Individual knowledge-sharing behavior	T1	0.704	0.877	0.882	0.512
	T2	0.703			
	T3	0.672			
	T4	0.659			
	T5	0.647			
	T6	0.637			
	T7	0.615			
	T8	0.632			
	T9	0.678			
	T10	0.606			
Knowledge-sharing behavior of others	Y1	0.693	0.929	0.859	0.532
	Y2	0.682			
	Y3	0.679			
	Y4	0.663			
	Y5	0.650			
	Y6	0.645			
	Y7	0.636			
	Y8	0.612			
Appreciation by others for those who share	Z1	0.711	0.920	0.825	0.574
	Z2	0.661			
	Z3	0.638			
	Z4	0.630			
	Z5	0.691			
	Z6	0.672			
	Z7	0.651			
	Z8	0.760			
	Z9	0.777			
	Z10	0.721			
	Z11	0.689			
	Z12	0.698			
	Z13	0.618			
Group performance	X1	0.894	0.811	0.804	0.573
	X2	0.892			
	X3	0.727			
	X4	0.708			
The individual status of the sharer	V1	0.892	0.906	0.795	0.795
	V2	0.825			
	V3	0.717			
	V4	0.723			
	V5	0.744			
	V6	0.703			
	V7	0.787			

### 4.3. Model fitness test

**Figure 2:**
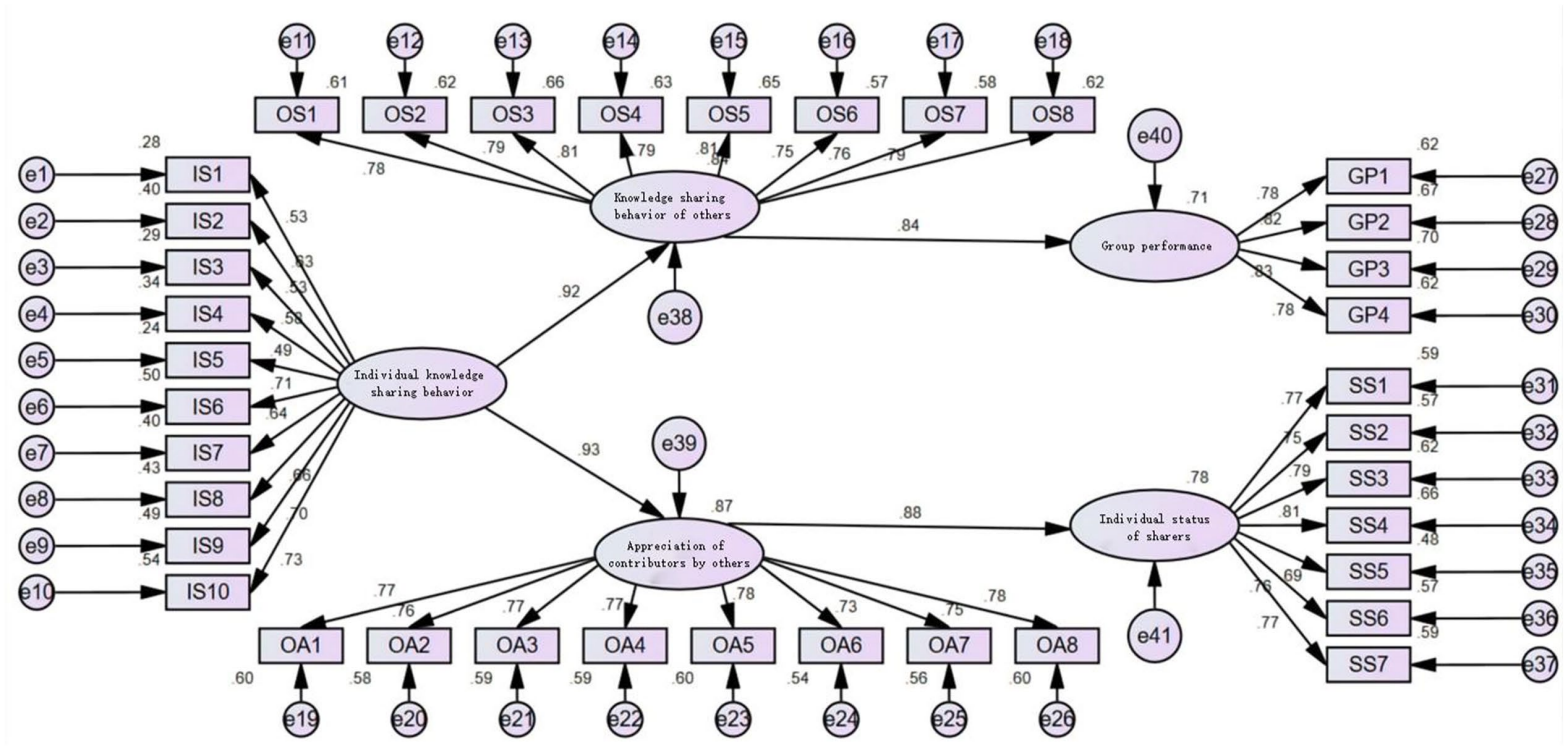
Structural equation model diagram.

To ensure that the data had a good fit, we conducted a validated factor analysis on five constructs: personal knowledge-sharing behavior (I.S.), others’ knowledge-sharing behavior (O.S.), others’ appreciation of sharers (O.A.) and group performance (G.P.), and sharers’ status (S.S.). The results of the validated factor analysis are shown in Table 3, where the five-factor model fits better (χ^2^/pdf = 2.100 < 3, CFI = 0.945 > 0.9, TLI = 0.940 > 0.9, RMSEA = 0.047 < 0.08, SRMR = 0.037 < 0.08), indicating that the five-factor model fits significantly better than the other competing models. Therefore, the five constructs of this study have good discriminant validity. The results of the analysis by Harman’s one-way test revealed that the factor with the most significant amount of explanation for EFA extraction explained a total of 0.17 of the variance (KMO = 0.978, *p* < 0.001), indicating that the standard method bias issue was at a manageable level of impact on this study. The model was tested for fitness using AMOS21; the test results are shown in Table 4. The results show that the absolute fitness index, value-added fitness index, and parsimony fitness index all exceeded the threshold values, indicating that the study model (Figure 2) has good explanatory power.

**Table 4:** Results of discriminant validity tests for each variable.

Models	*χ^2^*	*df*	*χ^2^ /df*	CFI	TLI	RMSEA	SRMR
The five-factor model (IS, OS, OA, GP, SS)	1299.640	619	2.100	0.945	0.940	0.047	0.037
Four-factor model (IS, OS, OA, GP + SS)	1339.577	623	2.150	0.942	0.938	0.048	0.038
Three-factor model (IS, OS+OA, GP + SS)	1598.865	626	2.554	0.921	0.916	0.056	0.041
Two-factor model (IS, OS+OA + GP + SS)	2075.649	628	3.305	0.882	0.875	0.068	0.047
One-factor model (IS+OS+OA + GP + SS)	2284.888	629	3.633	0.865	0.857	0.073	0.050

### 4.4. Hypothesis testing

As shown in Figure 2 and Table 5, individual knowledge-sharing behavior has a significant positive effect on others’ knowledge-sharing behavior (β = 0.918, *p* < 0.001), and hypothesis H1 holds. Personal knowledge-sharing behavior has a significant positive effect on others’ appreciation of the sharer (β = 0.933, *p* < 0.001), and hypothesis H3 holds. Also, it is worth noting that in the second half of the path in the model: knowledge sharing behavior of others has a significant positive effect on group performance (β = 0.844, *p* < 0.001), and appreciation of sharers by others has a significant positive effect on the individual status of sharers (β = 0.884, *p* < 0.001). Thus, as far as the direct path is concerned, the present model is supported by the data in all cases. A foundation was laid for subsequent tests of mediating effects.

**Table 5:** Results of model path analysis.

Paths	Standardized Parameter estimates	Standard error	*T*-value	Value of *p*
Individual knowledge-sharing behavior → Knowledge-sharing behavior of others	0.918	0.126	11.593	0.000
Individual knowledge-sharing behavior → Appreciation of the sharer by others	0.933	0.132	11.636	0.000
Knowledge sharing behavior of others → group performance	0.844	0.049	16.182	0.000
Appreciation of the sharer by others → Individual status of the sharer	0.884	0.050	16.427	0.000

### 4.5. Hypothesis testing

The study results have been shown to require 1,000 iterations of the original sample in the Bootstrap intermediate effects test. Suppose the Bootstrap mediation effect test results indicate that the Bootstrap test CI does not contain a value of 0. In that case, the indirect effect begins to operate. In this study, the method of calculating the mediating effect Bootstrap 95% CI based on 1,000 times of sample sampling for the mediating effect test, as seen above, the direct paths all passed the validation of the data. Thus, the two mediated paths are tested separately here. The effect test was performed using the bias-corrected percentile bootstrap method with 5,000 bootstrap replicate samples (Table 6).

**Table 6:** Results of model path analysis.

Intermediary effect path	Amount of effect	*T*-value	*p*-Value	Bootstrap 95% confidence interval	
				Lower limit	Upper limit
Individual knowledge-sharing behavior → others’ knowledge-sharing behavior → group performance	0.775	24.219	0.000	0.698	0.842
Individual knowledge-sharing behavior → Appreciation of the sharer by others → Individual status of the sharer	0.825	22.297	0.000	0.761	0.880

The above table shows that the 95% interval of the mediating effect of others’ knowledge-sharing behavior between individual knowledge-sharing behavior and group performance is (0.698, 0.842), with a significant mediating effect and an effect size of 0.775, and hypothesis H2 holds. Similarly, the 95% interval of the mediating effect of others’ appreciation of the sharer between individual knowledge-sharing behavior and the individual status of the sharer is (0.761, 0.880), with a significant mediating effect and an effect size of 0.825, and hypothesis H4 holds.

## 5. Discussion

### 5.1. Performance impact of knowledge sharing on groups

The performance impact caused by knowledge sharing in groups is undoubtedly an important consideration in how group members achieve effective communication and action (Huang et al., 2010; Ballesteros-Rodriguez et al., 2022). In this study, through a survey of 497 college students in six universities in China, we found that individual college students’ knowledge-sharing behaviors enhance group performance by influencing others’ sharing types. Discussing knowledge-sharing behaviors in the group space in depth helps group members to broaden their cognitive limits, change their perceptions, and enhance the acceptance and exchange of information (Huysman and Wulf, 2006; Huang et al., 2014). Knowledge sharing allows members to get mutual listening, active discussion, and effective communication, which leads to meaningful solutions and helps to shorten the time of communication effectively, dissemination of knowledge and information and can be rewarding from short-term benefit considerations (Huber, 2001; Cabrera and Cabrera, 2005). Therefore, scholars believe that knowledge sharing significantly enhances group performance regardless of the type of organization (e.g., business, university, government) (Wu and Lee, 2020; Choi et al., 2021).

It is important to note that although organization type does not change the effect of knowledge sharing on group performance. However, differences in organizational types can lead to different paths of the effect of organizational knowledge sharing on group performance. In the existing studies, scholars have more often conducted research at the enterprise level, arguing that corporate knowledge-sharing behavior is more based on corporate order and norms and that to improve teamwork efficiency, knowledge-sharing mechanisms must also be established to achieve performance improvement by sharing experiences and exchanging innovative ideas with each other (Wu and Lin, 2013; Choi et al., 2021; Kim, 2021). Individual knowledge sharing in enterprises is more of a compulsion. It does not drive others to share knowledge to enhance group performance. For example, in some enterprises, thematic activities are organized, including seminars, forums, and sharing sessions, where professionals and enterprise leaders share real-world experiences, thus allowing other employees to participate and enhance team performance actively. However, in university organizations, unlike enterprises, knowledge sharing among university students is unique. Learners can collect other people’s knowledge and share fantastic views through Internet platforms such as social software and knowledge-sharing forums, or activities of discussion and sharing, and stitch together new concepts with fragmented small knowledge and information, thus promoting each other’s thinking and interaction in this way to achieve the efficient promotion of learning and progress, and thus improving the group performance of learners (Pea, 2006; Pigg, 2014). Individual knowledge sharing among college students leads to knowledge sharing among others, which more effectively enhances group performance.

### 5.2. Impact of knowledge sharing behavior on individual social status

This study found that knowledge-sharing behaviors of college students can improve the social status of individuals, and the improvement of individuals’ social status is related to the degree of knowledge-sharing behaviors. Knowledge-sharing behavior can help individuals better integrate into society, enhance their social influence, and thus improve their social status (Bagherzadeh et al., 2019; Tintori et al., 2021). Currently, in the actual practice of student management in universities and colleges, due to the constraints of traditional concepts (Sun, 2021; Tao et al., 2021), limitations of the educational system (Ugnich et al., 2019), lack of professional training and exchange opportunities (Halberstadt et al., 2019), and the influence of organizational culture and work climate (Al-Husseini et al., 2019; Al-Kurdi et al., 2020), resulting in a general lack of knowledge management perceptions yet. Many leaders, teachers, and even student management workers in colleges and universities think that student management is practical work, mainly relying on accumulating work experience but not on establishing the concept of “knowledge in student management” (Kim, 2020). Due to the lack of rational understanding of knowledge in student management in colleges and universities, the quality and level of student management in colleges and universities are affected by the lack of accumulation of knowledge in student management in colleges and universities (Spence, 2019). Therefore, the majority of student management workers should first establish the concept of knowledge in college student management to fully understand the summary and analysis of students’ characteristics, the application of management theories, and the learning and summary of work experience in work practice are all knowledge in student management. Specifically, the concept of knowledge in student management in higher education refers to student management practitioners’ awareness and understanding of the importance and role of knowledge in enhancing the quality of management and advancing the social status of individual students (Bridgstock, 2009; Jackson, 2016). It emphasizes comprehensive knowledge, practical knowledge, and a culture of learning and sharing. First, knowledge in student management in higher education includes not only disciplinary knowledge but also comprehensive knowledge across disciplines (Mansilla and Duraising, 2007; Spelt et al., 2009). This includes but is not limited to knowledge in education, psychology, sociology, leadership, organizational management, and other related fields. Student management practitioners must integrate this knowledge to understand and respond to students’ needs and problems comprehensively. Second, knowledge in university student management is closely integrated with practice, emphasizing acquiring knowledge through accumulating and summating practical work experience (Gill, 2009). Student management workers should accumulate and apply the lessons learned in practice through interaction, observation, and reflection with students in order to improve management effectiveness. Finally, knowledge in university student management emphasizes a culture of learning and knowledge sharing (Al-Husseini et al., 2019; Al-Kurdi et al., 2020). Student management workers should be proactive in learning and updating their knowledge and continuously improve their professionalism and knowledge by attending training, seminars, and professional exchanges. At the same time, they should also encourage and support knowledge sharing and exchange within the team to promote mutual learning and growth of experiences. Only in this way can they better enhance the individual students’ social status through knowledge sharing.

Knowledge-sharing behaviors can also help individuals access more resources and opportunities, thus improving their social status (Sharma et al., 2021). Based on others’ appreciation of the sharer, there is an important mediating role between individual knowledge-sharing behaviors and individual social status. Especially in the group of college students, knowledge-sharing activities among individuals are beneficial to improve their social influence and social status. College students’ knowledge-sharing behaviors are mainly realized through social media and knowledge-sharing forums. Other Internet platforms and encouraging each other’s knowledge-sharing behaviors can improve individuals’ influence (Corvello et al., 2020) and recognition and are often appreciated and praised by others (Moghavvemi et al., 2017). According to today’s social values, ever-innovative knowledge-sharing behaviors can receive more recognition. Newer thinking skills can help college students better understand what they are learning (Hosen et al., 2021) and enhance their social status.

In contrast, the social status enhancement brought by corporate knowledge-sharing behaviors is relatively simple. Corporate knowledge sharing mostly has a more specialized character, and continuously accumulating years of knowledge and actively and effectively promoting the current area of expertise can help corporate employees gain social recognition more efficiently and gain a significant increase in social status. It can better meet the need for effective teamwork (Ben Zammel and Najar, 2022). Therefore, appreciation of the sharer by others can play an essential role as a glue between individual knowledge-sharing behavior and individual social status. Effective collaboration and social status enhancement can be achieved with the role of both parties, in both university student groups and corporate groups.

## 6. Limitations and future directions

This study reveals the mechanism of “others’ knowledge-sharing behavior” and “others’ appreciation of the sharer” in college students’ “knowledge-sharing behavior” on “group performance” and “individual social status.” It provides essential theoretical guidance for organizational knowledge management and cultivating college students’ learning abilities. However, there are still some limitations. First, in terms of investigating the selection of colleges and universities, this study only selected six representative colleges and universities in Guangdong Province, China. However, in China, more than 3,000 colleges and universities are distributed all over China. Due to the limitations of the geographical environment, the results may be affected to a certain extent. In future research, we will consider expanding the scope of the survey and conducting a sample survey for all Chinese universities. Secondly, regarding survey objects, this study only surveyed undergraduates in colleges and universities and did not investigate knowledge-sharing behaviors such as masters and doctors. Students at different stages have different quantities and qualities of knowledge, with apparent educational differences. In future research, this study will investigate scholars at different levels of education to compare the impact of differences in education further. In addition, we will clarify whether any knowledge shared by students participating in the study is considered appropriate to improve individual or group performance or whether criteria are involved and needed to select relevant, sound, or necessary knowledge from irrelevant knowledge. Finally, This study only focuses on the group performance and individual social status of college students and has yet to explore other characteristics, such as interpersonal trust and psychological characteristics. These aspects may be addressed by further research work in the future.

## 7. Conclusion

Knowledge-sharing behavior plays a vital role in driving the effectiveness of organizations and societies, serving as a valuable means to acquire new information and insights. Its successful implementation is closely associated with enhancing both group performance and individual social status. Effective knowledge sharing benefits not only the individuals engaging in it but also the overall group dynamics.

This study specifically focuses on investigating the knowledge-sharing behaviors of college students, examining the impact on individual social status and group performance. Additionally, the study explores the mediating role of others’ appreciation and knowledge sharing behaviors in this context. By employing advanced data analysis software, the following significant conclusions have been drawn: (1) Knowledge-sharing behaviors have a substantial positive influence on both group performance and individual social status. Higher education institutions should adopt appropriate measures to encourage students to actively participate in knowledge sharing activities. By doing so, not only can the performance of these institutions be improved, but also the social status of individuals within the educational community. (2) The appreciation shown by others plays a partially mediating role in the relationship between individual knowledge-sharing behavior and group outcomes. This means that when individuals receive appreciation from others for their sharing behavior, it not only positively reinforces their inclination to share knowledge but also contributes to their own social status concerns. This highlights the significance of creating a supportive environment where individuals recognize and value the knowledge contributions of their peers. (3) Individuals can make appropriate adjustments to their own knowledge-sharing behavior based on the observed behaviors of others. When individuals perceive a culture of active knowledge sharing among their peers, they are more likely to align their behavior accordingly, leading to improved group performance. This highlights the importance of promoting a collaborative and knowledge-sharing culture within college student groups. In conclusion, this research emphasizes the importance of knowledge sharing in higher education settings and its significant impact on both individual and group outcomes. It provides valuable insights for higher education institutions to devise strategies that encourage and facilitate knowledge sharing, ultimately enhancing organizational performance and the social status of individuals within the academic community.

## Data availability statement

The original contributions presented in the study are included in the article/supplementary material, further inquiries can be directed to the corresponding authors.

## Ethics statement

Ethical review and approval was not required for the study on human participants in accordance with the local legislation and institutional requirements. The patients/participants provided their written informed consent to participate in this study.

## Author contributions

All authors listed have made a substantial, direct, and intellectual contribution to the work and approved it for publication.

## Funding

This study was supported by the Guangdong Province Education Science Planning Project (Higher Education Special): 2022GXJK206; Ideological and Political Education Research Project of “One Hundred Youth Research Projects Funding Program,” Guangdong Medical University, Digital Twin and the Interinfiltration of Virtual and Real: A Study on the Logical Mechanism and Risk Prevention: 2022.

## Conflict of interest

The authors declare that the research was conducted in the absence of any commercial or financial relationships that could be construed as a potential conflict of interest.

## Publisher’s note

All claims expressed in this article are solely those of the authors and do not necessarily represent those of their affiliated organizations, or those of the publisher, the editors and the reviewers. Any product that may be evaluated in this article, or claim that may be made by its manufacturer, is not guaranteed or endorsed by the publisher.

## References

[ref1] Al-Husseini S. El Beltagi I. Moizer J. (2019). Transformational leadership and innovation: the mediating role of knowledge sharing amongst higher education faculty. Int. J. Leadersh. Educ. 24, 670–693. doi: 10.1080/13603124.2019.1588381

[ref2] Al-Kurdi O. F. El-Haddadeh R. Eldabi T. (2020). The role of organisational climate in managing knowledge sharing among academics in higher education. Int. J. Inf. Manag. 50, 217–227. doi: 10.1016/j.ijinfomgt.2019.05.018

[ref3] Bagherzadeh M. Markovic S. Cheng J. Vanhaverbeke W. (2019). How does outside-in open innovation influence innovation performance? Analyzing the mediating roles of knowledge sharing and innovation strategy. IEEE Trans. Eng. Manag., 67, 740–753.

[ref4] Ballesteros-Rodriguez J. L. De Saa-Perez P. Garcia-Carbonell N. Martin-Alcazar F. Sanchez-Gardey G. (2022). The influence of team members' motivation and leaders' behavior on scientific knowledge sharing in universities. Int. Rev. Adm. Sci. 88, 320–336. doi: 10.1177/0020852320921220

[ref5] Ben Zammel I. Najar T. (2022). Nexus between technological capital, organizational structure and knowledge sharing in organizational restructuring initiatives. VINE J. Inform. Knowledge Manage. Syst. 248898821. doi: 10.1108/VJIKMS-09-2021-0191

[ref6] Bridgstock R. (2009). The graduate attributes we’ve overlooked: enhancing graduate employability through career management skills. Higher Educ. Res. Dev. 28, 31–44. doi: 10.1080/07294360802444347

[ref7] Cabrera E. F. Cabrera A. (2005). Fostering knowledge sharing through people management practices. Int. J. Hum. Resour. Manag. 16, 720–735. doi: 10.1080/09585190500083020

[ref8] Calvó-Armengol A. Jackson M. O. (2009). Like father, like son: social network externalities and parent-child correlation in behavior. Am. Econ. J. 1, 124–150. doi: 10.1257/mic.1.1.124

[ref9] Cao Z. L. (2017). A study on the relationship between absorptive capacity, knowledge sharing and innovative behavior of college students. J. High. Educ. 15, 37–39.

[ref10] Chen Y. Jiang Y. J. Tang G. Cooke F. L. (2018). High-commitment work systems and middle managers' innovative behavior in the Chinese context: the moderating role of work-life conflicts and work climate. Hum. Resour. Manag. 57, 1317–1334. doi: 10.1002/hrm.21922

[ref11] Chen P. T. Kuo S. C. (2017). Innovation resistance and strategic implications of enterprise social media websites in Taiwan through knowledge sharing perspective. Technol. Forecast. Soc. Chang. 118, 55–69. doi: 10.1016/j.techfore.2017.02.002

[ref12] Chen W. Zhu X. Sun S. Liao S. Guo Z. (2021). The impact of Employees' psychological capital on innovative work behavior: the chain mediating effect of knowledge donating and knowledge collecting. Front. Psychol. 12:761399. doi: 10.3389/fpsyg.2021.761399, PMID: 34970192 PMC8712854

[ref13] Cheruvelil K. S. Soranno P. A. Weathers K. C. Hanson P. C. Goring S. J. Filstrup C. T.. (2014). Creating and maintaining high-performing collaborative research teams: the importance of diversity and interpersonal skills. Front. Ecol. Environ. 12, 31–38. doi: 10.1890/130001

[ref14] Choi Y. R. Kwon D. Y. Chang S. O. (2021). The development and effectiveness of a web-based emergency management educational program for long-term care facility Interprofessional practitioners. Int. J. Environ. Res. Public Health 18:12671. doi: 10.3390/ijerph182312671, PMID: 34886397 PMC8657401

[ref15] Corvello V. Chimenti M. C. Giglio C. Verteramo S. (2020). An investigation on the use by academic researchers of knowledge from scientific social networking sites. Sustainability 12:9732. doi: 10.3390/su12229732

[ref16] Dirks K. T. Skarlicki D. P. (2004). “Trust in leaders: existing research and emerging issues” in Trust and distrust in organizations: Dilemmas and approaches. eds. Kramer R. M. Cook K. S., vol. 7 (New York: Russell Sage Foundation), 21–40.

[ref17] Gan C. M. Wang W. J. Tian P. (2012). Research on psychological inducements of knowledge exchange and sharing in the academic blog. J. Libr. Sci. 38, 91–99.

[ref18] Gill A. (2009). Knowledge management initiatives at a small university. Int. J. Educ. Manag. 23, 604–616. doi: 10.1108/09513540910990834

[ref19] Goh S. C. (2002). Managing effective knowledge transfer: an integrative framework and some practice implications. J. Knowl. Manag. 6, 23–30. doi: 10.1108/13673270210417664

[ref20] Guo J. (2022). The significance of green entrepreneurial self-efficacy: mediating and moderating role of green innovation and green knowledge sharing culture. Front. Psychol. 13:1001867. doi: 10.3389/fpsyg.2022.1001867, PMID: 36211875 PMC9539384

[ref21] Halberstadt J. Timm J.-M. Kraus S. Gundolf K. (2019). Skills and knowledge management in higher education: how service learning can contribute to social entrepreneurial competence development. J. Knowl. Manag. 23, 1925–1948. doi: 10.1108/jkm-12-2018-0744

[ref22] Hosen M. Ogbeibu S. Giridharan B. Cham T. H. Lim W. M. Paul J. (2021). Individual motivation and social media influence on student knowledge sharing and learning performance: evidence from an emerging economy. Comput. Educ. 172:104262. doi: 10.1016/j.compedu.2021.104262

[ref23] Huang T. T. Chen L. Stewart R. A. (2010). The moderating effect of knowledge sharing on the relationship between manufacturing activities and business performance. Knowledge Manag. Res. Pract. 8, 285–306. doi: 10.1057/kmrp.2010.21

[ref24] Huang X. Hsieh J. P. A. He W. (2014). Expertise dissimilarity and creativity: the contingent roles of tacit and explicit knowledge sharing. J. Appl. Psychol. 99, 816–830. doi: 10.1037/a0036911, PMID: 24820927

[ref25] Huber G. P. (2001). Transfer of knowledge in knowledge management systems: unexplored issues and suggested studies. Eur. J. Inf. Syst. 10, 72–79. doi: 10.1057/palgrave.ejis.3000399

[ref26] Hung S. Y. Durcikova A. Lai H. M. Lin W. M. (2011). The influence of intrinsic and extrinsic motivation on individuals' knowledge-sharing behavior. Int. J. Hum. Comput. Stud. 69, 415–427. doi: 10.1016/j.ijhcs.2011.02.004

[ref27] Huysman M. Wulf V. I. T. (2006). To support knowledge sharing in communities, towards a social capital analysis. J. Inf. Technol. 21, 40–51. doi: 10.1057/palgrave.jit.2000053

[ref28] Jackson D. (2016). Developing pre-professional identity in undergraduates through work-integrated learning. High. Educ. 74, 833–853. doi: 10.1007/s10734-016-0080-2

[ref29] Kim T. (2020). Becoming skillful leaders: American school principals' transformative learning. Educ. Manage. Admin. Lead. 48, 353–378. doi: 10.1177/1741143218802596

[ref30] Kim S. L. (2021). Supervisor knowledge sharing and employee knowledge sharing: the moderating roles of learning goal orientation and affective organizational commitment. Sustainability 13:4176. doi: 10.3390/su13084176

[ref31] Lee J. N. (2001). The impact of knowledge sharing organizational capability and partnership quality on is outsourcing success. Inf. Manag. 38, 323–335. doi: 10.1016/S0378-7206(00)00074-4

[ref32] Leijen Ä. Allas R. Toom A. Husu J. Marcos J. J. M. Meijer P. (2014). Guided reflection for supporting the development of student teachers’ practical knowledge. Procedia Soc. Behav. Sci. 112, 314–322. doi: 10.1016/j.sbspro.2014.01.1170

[ref33] Li C. Li H. Suomi R. Liu Y. (2022). Knowledge sharing in online smoking cessation communities: a social capital perspective. Internet Res. 32, 111–138. doi: 10.1108/INTR-03-2020-0113

[ref34] Liao L. F. (2008). Knowledge-sharing in R & D departments: a social power and social exchange theory perspective. Int. J. Hum. Resour. Manag. 19, 1881–1895. doi: 10.1080/09585190802324072

[ref35] Lin H. F. (2007). Effects of extrinsic and intrinsic motivation on employee knowledge sharing intentions. J. Inf. Sci. 33, 135–149. doi: 10.1177/0165551506068174

[ref36] Mansilla V. B. Duraising E. D. (2007). Targeted assessment of students’ interdisciplinary work: an empirically grounded framework proposed. J. High. Educ. 78, 215–237. doi: 10.1080/00221546.2007.11780874

[ref37] Meng H. (2017). Research on the influencing factors of individual knowledge-sharing behavior in virtual relational communities. Fujian: Huaqiao University. Available at: https://kns.cnki.net/kcms2/article/abstract?v=3uoqIhG8C475KOm_zrgu4lQARvep2SAk-6BvX81hrs37AaEFpExs0I6ILjl6xC7xApzwn8EP6pJWneltLLahofeHWy14zh7D&uniplatform=NZKPT

[ref38] Moghavvemi S. Sharabati M. Paramanathan T. Rahin N. M. (2017). The impact of perceived enjoyment perceived reciprocal benefits and knowledge power on students' knowledge sharing through Facebook. Int. J. Manage. Educ. 15, 1–12. doi: 10.1016/j.ijme.2016.11.002

[ref39] Nguyen T. M. (2020). A review of two psychological models in knowledge sharing: current trends and future agenda. VINE J. Inform. Knowledge Manag. Syst. 51, 533–549. doi: 10.1108/vjikms-12-2019-0206

[ref40] Pea R. D. (2006). “Video-as-data and digital video manipulation techniques for transforming learning sciences research, education, and other cultural practices” in The international handbook of virtual learning environments. eds. Weiss J. Nolan J. Hunsinger J. Trifonas P. (Dordrecht: Springer), 1321–1393.

[ref41] Pereira V. Mohiya M. (2021). Share or hide? Investigating positive and negative employee intentions and organizational support in the context of knowledge sharing and hiding. J. Bus. Res. 129, 368–381. doi: 10.1016/j.jbusres.2021.03.011

[ref42] Pigg S. (2014). Coordinating constant invention: social Media's role in distributed work. Tech. Commun. Q. 23, 69–87. doi: 10.1080/10572252.2013.796545

[ref43] Razak N. A. Pangil F. Zin M. L. M. Yunus N. A. M. Asnawi N. H. (2016). Theories of knowledge sharing behavior in business strategy. Procedia Econ. Finance 37, 545–553. doi: 10.1016/s2212-5671(16)30163-0

[ref44] Razmerita L. Kirchner K. Nielsen P. (2016). What factors influence knowledge sharing in organizations? A social dilemma perspective of social media communication. J. Knowl. Manag. 20, 1225–1246. doi: 10.1108/jkm-03-2016-0112

[ref45] Sharma A. Bodger C. Elahi A. Halbich R. Jyoti H. Kennedy R.. (2021). Shared learning from the implementation of a technical leadership program. Sustainability 13:6433. doi: 10.3390/su13116433

[ref46] Spelt E. J. H. Biemans H. J. A. Tobi H. Luning P. A. Mulder M. (2009). Teaching and learning in interdisciplinary higher education: a systematic review. Educ. Psychol. Rev. 21, 365–378. doi: 10.1007/s10648-009-9113-z

[ref47] Spence C. (2019). “Judgement” versus “metrics” in higher education management. High. Educ. 77, 761–775. doi: 10.1007/s10734-018-0300-z

[ref48] Stupnisky R. H. Brcka Lorenz A. Yuhas B. Guay F. (2018). Faculty members' motivation for teaching and best practices: testing a model based on self-determination theory across institution types. Contemp. Educ. Psychol. 53, 15–26. doi: 10.1016/j.cedpsych.2018.01.004

[ref49] Sun Y. (2021). “The current situation and improvement suggestions of information management and Service in Colleges and Universities—Investigation of Xianyang City, Western China” in The 10th international conference on computer engineering and networks. CENet 2020. Advances in intelligent systems and computing. eds. Liu Q. Liu X. Shen T. Qiu X., vol. 1274 (Singapore: Springer)

[ref50] Tao A. Feng Z. X.. (2021) Evaluation research on online and offline blended teaching of organic chemistry based on logistic model. 2021 2nd international conference on education, knowledge and information management (ICEKIM), Xiamen, China, 112–115

[ref51] Tintori A. Ciancimino G. Giovanelli G. Cerbara L. (2021). Bullying and cyberbullying among Italian adolescents: the influence of psychosocial factors on violent behaviours. Int. J. Environ. Res. Public Health 18:1558. doi: 10.3390/ijerph18041558, PMID: 33562132 PMC7915616

[ref52] Ugnich E. Meskhi B. Ponomareva S. (2019). E-learning in higher inclusive education: needs, opportunities and limitations. Int. J. Educ. Manag. 33, 424–437. doi: 10.1108/ijem-09-2018-0282

[ref53] Wang Z. H. A. (2020). Review of domestic research on knowledge sharing in social media environment. Digital Library Forum 11, 63–72.

[ref54] Wang S. H. Xu B. Peng J. S. (2013). The influence of organizational climate perception on employee innovation behavior: mediating effect based on knowledge sharing intention. Sci. Res. Manag. 34, 130–135.

[ref55] Wu W. L. Lee Y. C. (2020). Do work engagement and transformational leadership facilitate knowledge sharing? A perspective of conservation of resources theory. Int. J. Environ. Res. Public Health 17:2615. doi: 10.3390/ijerph17072615, PMID: 32290352 PMC7177304

[ref56] Wu C. Lee C. Tsai L. (2012). Influence of creativity and knowledge sharing on performance. J. Technol. Manag. China 7, 64–77. doi: 10.1108/17468771211207358

[ref57] Wu L. W. Lin J. R. (2013). Knowledge sharing and knowledge effectiveness: learning orientation and co-production in the contingency model of tacit knowledge. J. Bus. Ind. Mark. 28, 672–686. doi: 10.1108/JBIM-04-2011-0050

[ref58] Wu S. Y. Wang W. T. Hsieh Y. H. (2021). Exploring knowledge sharing behavior in healthcare organizations: an integrated perspective of the empowerment theory and self-determination theory. Kybernetes 51, 2529–2553.

[ref59] Xu X. D. Wei Z. X. Zheng J. Y. (2021). A study on the impact of knowledge sharing among graduate students on their research performance. J. Manag. 18, 434–440.

[ref60] Zhang M. R. Jun Y. (2022). From group psychology to identity construction: a review of research on identity from multidisciplinary perspective. Guangdong Soc. Sci. 02, 202–214.

[ref61] Zhang Z. T. Wu Q. (2016). The influence of autonomous-controlled motivation on employee proactive behavior: the moderating role of organizational climate. J. South China Norm. Univ. 1, 123–131.

[ref62] Zhao S. S. (2012). An empirical study on the influence of performance appraisal politics on individual knowledge sharing behavior. Nankai Manag. Rev. 15, 150–160.

[ref63] Zhou X. (2022). Mental health self-efficacy as a moderator between the relationship of emotional exhaustion and knowledge hiding: evidence from music educational students. Front. Psychol. 13:979037. doi: 10.3389/fpsyg.2022.979037, PMID: 36160511 PMC9501982

